# What do we know about flares in spinal and pelvic girdle pain? A scoping review

**DOI:** 10.1186/s12891-026-09884-w

**Published:** 2026-04-28

**Authors:** Yu Kondo, Daiki Haga, Takashi Ariie, Haruki Ito, Takahiro Miki, Hiroshi Takasaki

**Affiliations:** 1 Graduate School of Health and Social Services, Saitama Prefectural University, Koshigaya, Japan; 2 Department of Rehabilitation, Sapporo Maruyama Orthopedic Hospital, Sapporo, Japan; 3 Department of Rehabilitation, Ageo Futatsumiya Clinic, Ageo, Japan; 4 Department of Physical Therapy, School of Health Sciences at Fukuoka, International University of Health and Welfare, Fukuoka, Japan; 5 Insight Lab, PREVENT Inc., Nagoya, Japan; 6 Department of Physical Therapy, Saitama Prefectural University, Koshigaya, Japan; 7 Center for Human Movement, Tokyo University of Technology, Tokyo, Japan

**Keywords:** Low back pain, Flare, Scoping review, Measurement, Risk factors

## Abstract

**Background:**

Flares in spinal and pelvic girdle pain, such as low back pain, can substantially impact quality of life; however, the understanding of definitions, assessment approaches, and associated factors remains limited. This scoping review mapped the literature on flare definitions, measurement tools, and reported risk factors in adults with spinal and pelvic girdle pain.

**Methods:**

We systematically searched five major databases (MEDLINE, EMBASE, CENTRAL, Web of Science, and CINAHL) to identify studies reporting on flares in spinal and pelvic girdle pain. Key data were extracted and synthesized thematically to map the current evidence and identify gaps.

**Results:**

Among the 9,880 articles screened, 16 studies satisfied the inclusion criteria, all focusing exclusively on low back pain. Although definitions of flares had shifted from pain-centered models to multidimensional frameworks incorporating functional and emotional impacts, consensus on specific domains remains lacking. Assessment tools, which primarily included numerical rating scales and single-item self-reports, were identified, but none had psychometric validation. Physical activity, sleep, and psychological factors were identified risk factors for flares; however, the available evidence was limited and inconsistent, with most studies having a high risk of bias and focusing on a narrow range of factors.

**Conclusions:**

This review highlights significant gaps in the understanding and assessment of flares in spinal and pelvic girdle pain. Future research should focus on establishing a consensus on flare domains, developing validated assessment tools, and conducting rigorous studies to identify a broader range of risk factors across spinal and pelvic girdle pain.

**Supplementary Information:**

The online version contains supplementary material available at 10.1186/s12891-026-09884-w.

## Introduction

Flares, acute exacerbations characterized by unpredictable fluctuations in intensity, frequency, and duration, have profound effects on individuals living with spinal and pelvic girdle pain, such as low back pain (LBP). LBP alone affects approximately 540 million people globally at any given time and 80% of adults experiencing LBP during their lifetime [1]. Its clinical course is variable rather than a single self-limited episode [2], and flares have been reported in both persistent and episodic conditions [3, 4]. In a primary care survey of chronic nonspecific LBP, approximately 51% of patients reported flares over the previous 6 months [4]. Aside from physical discomfort, flares reduce quality of life, work productivity, and mental health [4, 5]. Moreover, their unpredictability disrupts treatment regimens, necessitating frequent adjustments in therapeutic approaches, extending recovery periods, and escalating healthcare costs [6–8].

Although a thorough understanding of flares is extremely important for identifying effective treatment for spinal and pelvic girdle pain and educating patients on long-term preventive strategies through self-management, a large gap in current knowledge remains. First, understanding of the definitions used and domains assessed in existing research on flares in spinal and pelvic girdle pain remains limited. A 2018 systematic review addressing the definitions of flares across various musculoskeletal conditions found that very few studies have investigated the definitions of flares specifically for LBP and neck pain, highlighting a critical gap in the literature [9]. Second, studies have utilized diverse methods for assessing flares, with no consensus having been established, thereby complicating the comparison and synthesis of data across studies [4, 10–13]. Finally, evidence on risk factors and triggers of flares remains limited. A comprehensive understanding of these factors would certainly enable healthcare providers to estimate the risk of flares and consider strategies for their management. These gaps highlight the need for a comprehensive review that would consolidate existing knowledge and guide future research.

Therefore, this scoping review aimed to (1) identify definitions and domains of flares described in the literature on spinal and pelvic girdle pain; (2) summarize the tools and approaches used to assess flares and identify gaps in evidence on their measurement properties; and (3) summarize reported factors associated with flares, including potential risk factors and triggers. The findings are intended to inform priorities for future research on flare definitions, measurement, and associated factors in these conditions.

## Methods

This review was written in accordance with the Joanna Briggs Institute Manual for Evidence Synthesis [14]. Our results were reported according to the Preferred Reporting Items for Systematic Reviews and Meta-Analyses Extension for Scoping Reviews (PRISMA-ScR) (Additional file 1) [15]. The review protocol was uploaded to the Open Science Framework [16].

### Target populations

This review focused on studies involving adults aged 18 years and older with spinal and pelvic girdle pain. Eligible conditions included a broad spectrum of musculoskeletal conditions involving the spine and pelvic girdle [17, 18], such as nonspecific low back pain, idiopathic neck pain, whiplash-associated disorders, radiculopathy, scoliosis, spinal stenosis, herniated intervertebral discs, vertebral compression fractures, thoracic spine pain, and pelvic girdle pain. These criteria were applied irrespective of whether the participants had undergone any surgical interventions. Studies focusing on red-flag or serious pathologies, such as infection, malignancy, or inflammatory disease, were excluded. Studies in which symptoms were primarily attributable to non-musculoskeletal causes, including visceral referred pain, were also excluded.

### Concept

This review included studies reporting on flares in spinal and pelvic girdle pain, focusing specifically on those that provided explicit definitions or descriptions of flares. Moreover, studies reporting on tools and assessment methods used to evaluate flares were included, regardless of whether the reliability or validity of these tools and methods had been assessed. Furthermore, studies investigating risk factors and triggers associated with flares in spinal and pelvic girdle pain were considered. However, those that implied the occurrence of flare episodes without providing a clear definition or explicitly mentioning flares were excluded.

### Context

No restrictions were placed with regard to context.

### Types of sources

This review only included primary peer-reviewed studies published in English, regardless of research design. No time limit was imposed on the publication date of the studies.

### Search strategy

Two authors (YK and HI) collaboratively conducted a search of the following databases from inception to March 2025: MEDLINE, EMBASE, the Cochrane Central Register of Controlled Trials (CENTRAL), Web of Science, and CINAHL. A systematic search strategy was developed using the Medical Subject Headings (MeSH) terms and keywords related to flares in spinal and pelvic girdle pain generated from subject headings, examples of which are presented in Additional file 2. Additionally, the author manually searched the reference lists of the included studies and relevant reviews to identify any additional relevant studies.

### Study selection

Following the search, all identified citations were collated and uploaded into Rayyan software [19], and duplicates were removed. Two independent reviewers (YK and DH) then screened the titles and abstracts for assessment against the inclusion criteria for the review. Any disagreements between the reviewers were resolved through discussion. Subsequently, the full texts of potentially relevant studies that remained following the screening process were retrieved and comprehensively assessed against the inclusion criteria by the two independent reviewers. Any disagreements between the reviewers were again resolved through discussion. The reasons for exclusion were recorded.

### Data extraction

The two reviewers (YK and DH) independently extracted the data using an electronic form. The extracted data included specific details about the study characteristics (authors, year of publication, study design, country of origin, and objective); the characteristics of the study population (condition, number of participants, and the age and gender of participants); the definition or domain of flares in the study; tools and measures used to evaluate flares, including their validity and reliability; factors or triggers identified to have contributed to the occurrence of flares; and key findings associated with flares. We included both univariate analyses and multivariate analyses when extracting data on risk factors and triggers associated with flares. Various study designs were considered, including prospective cohort studies, case-crossover studies, cross-sectional studies, and qualitative studies. When multiple time points for risk factors were reported, the results with the strongest association with flares were selected. If the strongest association was not clearly indicated, data from the time point closest to the flare occurrence were selected. For studies using multiple definitions of flares, we prioritized those that presented the most comprehensive definition. Any disagreements between the two reviewers were resolved through discussion.

### Evaluation of methodological quality and measurement property

The methodological quality of studies reporting on the reliability and validity of the tools used to assess flares was determined by two independent reviewers (YK and DH) using the COnsensus‐based Standards for the selection of health Measurement INstruments (COSMIN) checklist [20]. This checklist covers nine measurement properties, including internal consistency, reliability, measurement error, content validity, structural validity, hypothesis testing, cross-cultural validity, criterion validity, and responsiveness. Each measurement property is rated on a four-point scale (excellent, good, fair, or poor), with the lowest score indicating the methodological quality of each property. Unreported items were marked as not reported. Any disagreements between the two reviewers were resolved through discussion.

Two independent reviewers (YK and DH) used the Quality in Prognosis Studies (QUIPS) tool to assess the methodological quality of studies reporting on risk factors associated with flares [21]. QUIPS, which had been recommended by Cochrane for evaluating the risk of bias (RoB) in prognostic studies, covers six domains, namely study participation, study attrition, prognostic factor measurement, outcome measurement, study confounding, and statistical analysis and reporting. Each domain is graded as having low, moderate, or high RoB. An overall RoB score was then assigned to each study based on the scores for all domains. If all six domains were rated as having a low RoB, the study was classified as having a low RoB. If one or more domains were rated as having a high RoB or ≥ 3 domains were rated as having a moderate RoB, the study was classified as having a high RoB. All studies that did not fall within these classifications were classified as having a moderate RoB [22]. Any disagreements between the two reviewers were resolved through discussion.

### Certainty of evidence

The certainty of evidence was determined using two modified Grading of Recommendations Assessment, Development and Evaluation (GRADE) approaches [23–26], one for measurement properties and another for risk factors. Two independent reviewers (YK and DH) conducted the assessments, with disagreements being resolved through discussion. The overall certainty of evidence was categorized as high, moderate, low, or very low. For measurement properties, the starting point was high-quality evidence. For risk factors, the starting point was determined based on the phase of the investigation: high for explanatory studies confirming independent associations or investigating prognostic pathways (phase 2–3) and moderate for predictive modeling studies or explanatory studies conducted in the earlier phase to generate hypotheses (phase 1).

For measurement properties, the certainty of evidence was evaluated using four domains (study limitations, inconsistency, indirectness, and imprecision). Study limitations were assessed using the COSMIN checklist, with the certainty of evidence being downgraded by one level for doubtful quality, two levels for inadequate quality, and three levels for extremely inadequate quality. For inconsistency, the certainty of evidence was downgraded by one level if heterogeneity partially affected the interpretation of the results and by two levels if heterogeneity substantially affected the interpretation of the results when the differences could not be explained by the study characteristics. For indirectness, the certainty of evidence was downgraded by one level when the findings did not directly apply to the population, setting, or outcomes of interest. For imprecision, the certainty of evidence was downgraded by one level when the total sample size was below 100 and by two levels when it was below 50.

For risk factors, the certainty of evidence was evaluated using five domains (i.e., study limitations, inconsistency, indirectness, imprecision, publication bias). Study limitations were assessed using the QUIPS tool and downgraded by one level for moderate or unclear RoB and by two levels for high RoB. For inconsistency, the certainty of the evidence was downgraded by one level when estimates varied in direction with minimal confidence interval overlap or when only one study was available. For indirectness, the certainty of evidence was downgraded by one level when the findings did not directly apply to the population, setting, or outcomes of interest. For imprecision, the certainty of evidence was downgraded by one level for wide confidence intervals including both protective and risk values, particularly with limited samples (< 100 cases for continuous outcomes or < 10 events per predictor for dichotomous outcomes). Publication bias was considered present by default given that negative results often remain unpublished in prognostic studies. Downgrading was waived only when the risk factor had been extensively studied across multiple phase 2–3 cohort studies.

### Data analysis and presentation

A narrative synthesis of the findings was conducted by organizing the results thematically and summarizing key insights. Our results are presented in tables and figures, accompanied by descriptive summaries, in accordance with the PRISMA guidelines for scoping reviews [15].

## Results

### Study selection

**Fig. 1:**
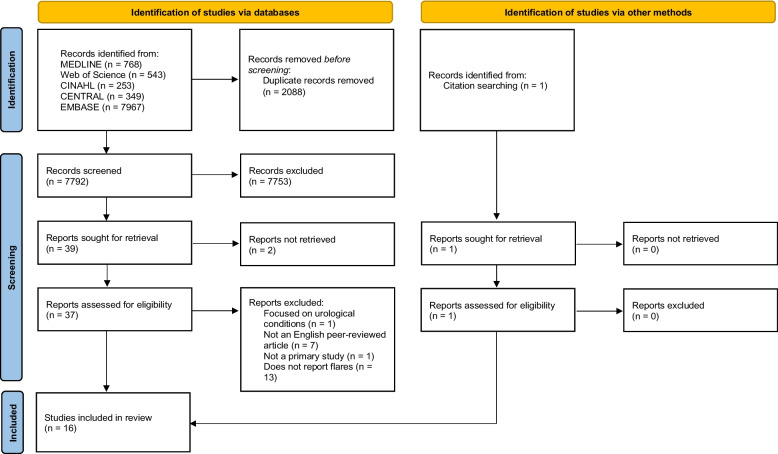
PRISMA flow diagram

The initial database search yielded a total of 9,880 studies. After removing duplicates and screening titles and abstracts, 37 studies were selected for full-text assessment. Through citation searching, one additional relevant article was identified, bringing the total number of full-text studies assessed to 38. After applying the inclusion and exclusion criteria, 16 studies were ultimately included in the current scoping review [3, 4, 11–13, 27–37]. Figure 1 depicts the PRISMA flow diagram for the selection process. The rate of agreement between the two independent reviewers (YK and DH) with regard to study inclusion was 99.6% during the initial screening phase and 92.1% during the full-text inspection phase.

### Study characteristics

The 16 included studies utilized various research designs: a prospective longitudinal design (seven studies) [3, 11–13, 27, 28, 37], qualitative cross-sectional design (four studies) [29–32], randomized controlled trial (two studies) [33, 34], and others (three studies, one each for a cross-sectional, mixed-methods, and one-arm intervention design) [4, 35, 36]. Most studies were conducted in Australia (seven studies) [12, 13, 27, 29–31, 35] and the United States (five studies) [3, 4, 11, 32, 33], with the remaining studies coming from Canada (two studies) [28, 36], China (one study) [37], and India (one study) [34]. The majority of the studies (13/16, 81.3%) focused on chronic LBP, whereas two studies examined acute LBP [3, 11], and one study specifically investigated acute and subacute lumbar radicular pain [37]. No studies specifically investigated pelvic girdle pain, neck pain, or other conditions. Sample sizes varied considerably, ranging from 17 to 621 participants, with mean ages ranging from 29.0 to 54.9 years. Most studies reported on the gender distribution of their participants, often showing a slight female predominance. Table 1 summarizes the characteristics of the included studies.

**Table 1:** Characteristics of the included studies

Authors, year of publication, country of origin	Study objective	Study design	Participant characteristics	Key findings related to flares
Coats et al., 2004, USA [33]	To assess the analgesic efficacy and tolerability of valdecoxib 40 mg/day by comparing it with placebo in the treatment of chronic LBP flares	RCT	Chronic LBP flares; 293 participants Age (mean ± SD): Valdecoxib group, 48.6 ± 13.3; Placebo group, 48.7 ± 12.6 Gender (M/F): Valdecoxib group, 67/81; Placebo group, 60/85	Valdecoxib reduced pain, improved function, and reduced disability in chronic LBP flare compared with placebo
Costa et al., 2021, Australia [27]	To compare self-reported flares with pain-defined flares and investigate the differences in physical and psychosocial features between these types of flares	Prospective, longitudinal case-crossover study	Chronic LBP; 126 participants Age (mean ± SD): 29 ± 9 Gender (M/F): 34/52	Self-reported and pain-defined flares showed different physical and psychosocial features, with poor sleep quality and morning pain increasing the odds of both flare types
Costa et al., 2021, Australia [12]	To identify risk factors for LBP flares and examine whether they differ based on the flare definition	Prospective, longitudinal case-crossover study	Chronic LBP; 126 participants Age (mean ± SD): 29 ± 9 Gender (M/F): 34/52	We investigated the risk factors for flares and found that these factors differ depending on whether flares are defined by pain alone or a broader, multidimensional definition
Costa et al., 2022, Australia [13]	To investigate whether objective measures of physical activity and sleep were associated with the risk of LBP flares and whether such an association differed according to the definition of flares	Prospective, longitudinal case-crossover study	Chronic LBP; 126 participants Age (mean ± SD): 29 ± 9 Gender (M/F): 34/52	Variations in physical activity and in-bed time were associated with the risk of LBP flare, with longer in-bed time increasing the risk of pain-defined flares. Sedentary behavior increased the risk of self-reported flares, whereas physical activity decreased such risk
Costa et al., 2019, Australia [35]	To develop a consensus definition for flares in LBP	Qualitative study using interviews, thematic analysis, and the Delphi process	Step 1: 130 individuals with LBP Step 2: 19 experts Step 3: 50 experts Step 4: 16 individuals with LBP	A consensus definition of LBP flare was developed, emphasizing a broad range of aspects beyond pain
Costa et al., 2019, Australia [29]	To investigate individual perspectives on triggers of LBP flares	Qualitative cross-sectional study	LBP; 130 participants Age (mean ± SD): 43.2 ± 12.05 Gender (M/F): 34/96	Participants mainly reported biomedical triggers (84.8%), such as movement and posture, with fewer noting nonbiomedical triggers (15.2%) like psychological state and weather
Dawu et al., 2024, China [37]	To determine whether specific physical activity or psychological stress factors are associated with different definitions of flares among people with lumbar radicular pain	Prospective, longitudinal case-crossover study	Acute or subacute lumbar radicular pain; 152 participants Age (mean ± SD): 47.5 ± 15.9 Gender (M/F): 81/71	Prolonged sitting, walking, and standing were significantly associated with pain-defined flares, while prolonged sitting, mental distress, and depressed mood were associated with non-pain-defined flares
Macedo et al., 2021, Canada [36]	To determine the feasibility of the back to living well program developed to prevent recurrences or flares in LBP	One-arm intervention study	LBP; 17 participants Age (mean ± SD): 54.9 ± 11.7 Gender (M/F): 8/9	Four participants experienced flare-ups over 23 weeks, with six of the eight flare-ups occurring during the 12-week intervention. The average pain increase was 2.6/10, and the median duration was 1 week (range: 1–6 weeks)
McGorry et al., 2000, USA [28]	To describe daily pain reporting by individuals with chronic and recurrent LBP, study how pain intensity and its episodic nature relate to disability and medication use	Prospective, longitudinal study	Chronic or recurrent LBP; 94 participants Age (mean ± SD): 44.2 ± 7.6 Gender (M/F): 50/44	Pain intensity affects disability; during flare-ups, disability and medication use were significantly greater
Muthukrishnan et al., 2010, India [34]	To compare the effects of core stabilization exercises and conventional physiotherapy on postural control in Chronic LBP	RCT	Chronic LBP; 30 participants Age (mean ± SD): Movement impairment group, 34.21 ± 8; Control impairment group, 36.21 ± 8 Gender (M/F): Movement impairment group, 9/6; Control impairment group, 8/7	Core stability exercises significantly improved postural control and ground reaction forces and significantly decreased flare-up recurrence
Setchell et al., 2017, Australia [30]	To understand how people with LBP conceptualize flare and whether an LBP flare is equivalent to an increase in pain	Qualitative cross-sectional study	LBP; 130 participants Age (mean ± SD): 43.2 ± 12.05 Gender (M/F): 34/96	Participants identified multiple aspects of flares beyond pain increase, including: functional limitations, difficulty alleviating symptoms, and emotional/cognitive changes. Among the included patients, 47% did not equate flare with pain increase
Suri et al., 2018, USA [11]	To determine whether physical activities trigger flare-ups of pain during the course of acute LBP	Prospective, longitudinal case-crossover study	Acute LBP; 48 participants Age (mean ± SD): 49.8 ± 13.2 Gender (M/F): 20/28	Prolonged sitting (> 6 h) and stress or depressive symptoms were identified as significant triggers of flares; physical activity was not identified as a consistent trigger
Suri et al., 2011, USA [3]	To characterize fluctuations in pain during acute LBP, to determine whether self-reported “flares” represent discrete periods of increased pain intensity; and to examine whether flare frequency was associated with back-related disability outcomes	Prospective, longitudinal case-crossover study	Acute LBP; 47 participants Age (mean ± SD): 49.7 ± 13.4) Gender (M/F): 27/20	A high frequency of flares was associated with a poor prognosis, with both pain intensity and flare frequency having been linked to increased disability and decreased physical function
Suri et al., 2012, USA [4]	To describe the prevalence and characteristics of flare-ups of chronic LBP among primary care patients and examine their associations with measures of pain severity and psychosocial factors	Cross-sectional study	Chronic LBP; 621 participants Age (mean ± SD): 47.9 ± 0.5 Gender (M/F): 297/324	Subjects with flare-ups experienced increased pain intensity, disability, opioid medication use, and psychosocial comorbidities
Tan et al., 2019, Australia [31]	To explore the changes in an individual’s life upon experiencing LBP flare-ups	Qualitative cross-sectional study	LBP; 130 participants Age (mean ± SD) 43.2 ± 12.05 Gender (M/F): 34/96	Flares triggered feelings of disablement, mood changes, reliance on passive coping strategies, and a lack of understanding from others, affecting social life, work, and daily activities
Young et al., 2011, USA [32]	To further the understanding of LBP recurrence and how to measure it and to examine how individuals with a history of LBP describe their back pain experiences	Qualitative cross-sectional study	LBP; 31 participants Age range from 20 to 60 years Gender (M/F): 17/14	Participants defined three states: “normal,” “flared-up,” and “attack,” highlighting the diverse and episodic nature of low back pain

*Abbreviations*: *LBP* low back pain, *RCT* randomized controlled trial, *M/F* Male/Female

### Definitions and domains of flares

Our scoping review identified 15 studies investigating the definitions and domains of flares in spinal and pelvic girdle pain [3, 4, 11–13, 27–34, 36]. These studies focused exclusively on LBP and lumbar radicular pain, with no study investigating other spinal and pelvic girdle pain. Table 2 summarizes the definitions of LBP flare found in the literature.

**Table 2:** Summary of flare definitions and assessment tools

Study	Definition/domain of flares	Measure used for assessing flares
McGorry et al., 2000 [28]	Flare-up was defined as 2 to 9 consecutive days of pain scores equal to or greater than two pain score units (on a scale of 0–10) above the participant’s median pain score throughout the 6-month period	NRS
Coats et al., 2004 [33]	VAS score ≥ 40 mm, a ≥ 10 mm increase in VAS score from the screening visit, > 1 point increased on the Patient’s Global Assessment scale	VAS and Patient’s Global Assessment scale
Muthukrishnan et al., 2010 [34]	Flare-ups were defined as an increase in pain intensity of at least two units on a 0–10 scale from baseline during the intervention period	NRS
Suri et al., 2011 [3]	A period of increased pain lasting at least 2 h, when the pain intensity is distinctly worse than it has been recently	Self-reported assessment of the presence and duration of current pain flare
Young et al., 2011 [32]	Flare-ups were characterized by an increased experience of pain but not intense enough to cause some additional activity limitations and participation restrictions	Not applicable
Suri et al., 2012 [4]	Flare-ups were characterized as back pain that is much worse than usual for days, weeks, or months at a time	Self-reported assessment of the presence and number of pain flares in the last six months
Setchell et al., 2017 [30]	Not predefined. The study identified four main themes related to flares: (1) increase in pain and other uncomfortable sensations; (2) increase in area, quality and/or duration of symptoms; (3) reduction in physical, cognitive and/or social functioning; and (4) negative psychological and/or emotional factors	Not applicable
Suri et al., 2018 [11]	The same definition as that used by Suri et al., 2011 [3]	Self-reported assessment of the presence and duration of current pain flare
Costa et al., 2019 [35]	A flare-up was defined as a worsening of one’s condition that lasts from hours to weeks, is difficult to tolerate, and generally impacts your usual activities and/or emotions	Not applicable
Tan et al., 2019 [31]	Not predefined. The study identified four main themes related to flare impact: (1) sense of disablement, (2) changes in mood, (3) use of coping strategies, and (4) lack of understanding from other people	Not applicable
Costa et al., 2021 [27]	Domain 1: Pain-defined flare, pain increase of two or more points on the 11-point NRS; above average pain on non-flare days Domain 2: Self-reported flare, an increase in pain or other related symptoms that lasts from hours to weeks and is difficult to settle. Mood changes and/or difficulty with your normal activities may also be present	NRS and self-reported assessment of flare occurrence
Costa et al., 2021 [12]	Same definition as that used Costa et al., 2021 [27]	NRS and self-reported assessment of flare occurrence
Macedo et al., 2021 [36]	Same definition as that used by Costa et al., 2019 [35]. Operationally defined as worsening of pain by ≥ 2 points on a 0–10 scale and activity limitation score ≥ 3 on a 1–5 scale	NRS and PROMIS
Costa et al., 2022 [13]	Same definition as that used by Costa et al., 2021 [27]	NRS and self-reported assessment of flare occurrence
Dawu et al., 2024 [37]	Domain 1: Pain-defined flares, periods of increased pain lasting at least 2 h, when pain intensity is distinctly worse than it has been recently Domain 2: Non-pain-defined flares, uncomfortable feelings such as fatigue, loss of function, or emotional/psychosocial fluctuations, without major fluctuations in pain intensity	NRS

*Abbreviations*: *VAS* visual analog scale, *NRS* numerical rating scale, *PROMIS* Patient-Reported Outcomes Measurement Information System

### Pain-centric definitions

Early definitions focused primarily on the intensity and duration of pain. In 1994, Von Korff defined a flare as “a period of pain lasting at least two hours in which the intensity of the pain is markedly worse than recently” [3, 11]. McGorry et al. [28] introduced a quantitative threshold, defining a flare as “a period of two to nine consecutive days during which the pain score is two or more points higher than the participant’s median pain score over a six-month period.” This two-point increase criterion had been adopted in several subsequent studies [12, 13, 27, 34, 36]. Suri et al. [4] defined a flare as “back pain that is much worse than normal for days, weeks, or months at a time,” thereby extending the short-term perspective to longer durations.

### Integration of functional impacts

Recognizing that flares involve more than just pain, researchers had started to incorporate functional limitations into their definitions. Indeed, Young et al. [32], who conducted focus groups with 31 participants, concluded that flares cause “some additional activity limitations and participation restrictions.” Macedo et al. [36] quantified this definition by operationalizing flares as increased pain (≥ 2 points on a 0–10 scale) coupled with activity limitation (≥ 3 points on a 1–5 scale using Patient-Reported Outcomes Measurement Information System item PI9).

### Multidimensional conceptualizations

Recent qualitative studies have provided deeper insights into the complexity of flares. After analyzing data from 130 individuals with LBP, Setchell et al. [30] identified themes such as increased pain and discomfort; expanded symptom areas; reduced physical, cognitive, and social functioning; and negative psychological impacts. Interestingly, nearly half of the participants (47%) did not associate flares solely with increased pain. After similarly studying 130 participants, Tan et al. [31] also identified themes like a sense of disablement, mood changes, coping strategies, and lack of understanding from others. Participants reported that flares negatively affected several aspects of their lives, including social interactions, work, and emotional well-being. Alarmingly, some even expressed thoughts of self-harm. Several studies have since distinguished between pain-defined flares and broader conceptualizations, including Costa et al. [12, 13, 27] comparing pain-defined and self-reported flares, and Dawu et al. [37] contrasting pain-defined and non-pain-defined flares in lumbar radicular pain.

### Consensus and standardization

To address the variability in definitions, Costa et al. [35] conducted a rigorous, multi-stage study involving patients and experts to develop the following definition of LBP flare: “A flare is a worsening of your condition that lasts from hours to weeks, is difficult to tolerate, and generally affects your usual activities and/or emotions.” This comprehensive definition encompasses not only pain but also the functional and emotional impacts over a variable time frame. Since its publication, this definition or its provisional version has been adopted in four studies published after 2019 [12, 13, 27, 36], reflecting a shift toward standardization in the field.

### Flare assessment tools

Our review identified eleven studies that evaluated LBP flares using various assessment tools [3, 4, 11–13, 27, 28, 33, 34, 36, 37] (Table 2). The Numerical Rating Scale (NRS), which was employed in seven studies, was the most commonly used tool [12, 13, 27, 28, 34, 36, 37]. The Visual Analog Scale (VAS), another measure of pain intensity, was used in one study [33]. Beyond pain intensity measures, the assessment of other dimensions was limited. Only two studies incorporated broader assessments: one study [36] used the Patient-Reported Outcomes Measurement Information System for activity limitation, while another study [37] employed multiple 11-point NRS to assess physical activity difficulty, fatigue, and depressed mood when evaluating non-pain-defined flares. The Patient’s Global Assessment Scale, which assesses the worsening of symptoms on a 5-point scale, was used in one study [33] as part of the flare criteria. Self-reported evaluations of current flare presence were used in six studies [3, 4, 11–13, 27]. For example, Suri et al. [4] used a self-reported assessment of the presence and number of pain flares over the last 6 months. However, none of the eleven studies provided information on the psychometric properties of these tools. Although we initially planned to evaluate the RoB of each tool using the COSMIN and apply the GRADE framework to assess the certainty of evidence, the lack of necessary psychometric data made both COSMIN and GRADE evaluations infeasible for these tools.

### Risk factors and triggers

Among the five studies that evaluated the risk factors and triggers for flares [11–13, 29, 37], four were quantitative studies, whereas one was a qualitative study. All quantitative studies used conditional logistic regression in case-crossover designs: two studies used univariate analysis [12, 13], whereas two studies used both univariate and multivariate analysis [11, 37]. The qualitative study used thematic analysis to explore their participants’ perspectives on flare triggers [29]. Most studies focused exclusively on LBP, with one study specifically examining lumbar radicular pain.

Table 3 presents the results of the RoB assessment for each study using the QUIPS tool. Notably, all four included studies were assessed as having a high overall RoB [11–13, 37]. Specifically, the domain “Study Confounding” was rated as having a high RoB in all four studies. The certainty of evidence evaluated using the GRADE framework is summarized in Additional file 3. Accordingly, the overall certainty of evidence for each risk factor was rated as very low, with the study limitation, inconsistency, imprecision, and publication bias being the main reasons for the downgrading of the certainty of evidence.

**Table 3:** Evaluation of the potential risk of bias with the Quality In Prognostic Studies (QUIPS) tool

Study	Study Participation	Study Attrition	Prognostic Factor	Outcome	Study Confounding	Analysis	Overall Risk of Bias
Costa et al., 2021 [12]	Low	Moderate	Moderate	Moderate	High	Low	High
Costa et al., 2022 [13]	Low	Moderate	Low	Moderate	High	Low	High
Dawu et al., 2024 [37]	Low	Low	High	Moderate	High	Low	High
Suri et al., 2018 [11]	Low	Moderate	High	Moderate	High	Low	High

### Physical activity and sedentary behavior

Prolonged sitting was consistently associated with increased flare risk across studies, with significant associations observed for both self-reported sitting for > 6 h (odds ratio [OR] of 4.2 [95% confidence interval: 1.9—9.1]) [11] and > 1 h (OR of 3.4 [1.8—6.2] [37]), and objectively measured sedentary time associated with increased flare risk after 1 day (OR of 1.03 [1.00—1.05]) [13]. Objectively measured standing time was associated with a reduced risk of flares after 1 day (OR of 0.97 [0.95—1.00]) [13], while self-reported prolonged standing showed no significant associations with flares within 24 h for both standing > 6 h (OR of 1.6 [0.2—17]) [11] and standing > 1 h (OR of 1.5 [0.8—3.0]) [37]). Neither objectively measured walking time (OR of 0.97 [0.92—1.02]) [13] nor self-reported prolonged walking (> half an hour) (OR of 1.0 [0.6—1.9]) [37] showed significant associations with flares within 24 h. Low-intensity activities (% metabolic equivalent [MET] < 1.4) were also associated with an increased risk of flares after 1 day (OR of 1.03 [1.00—1.05]), whereas activities with a high total MET were associated with a reduced risk of flares after 3 days (OR of 0.96 [0.91—1.00]) [13]. Leisure physical activity was not significantly associated with flares over the subsequent 3 days (OR of 1.16 [0.67—2.00]) [12]. Other specific activities, such as prolonged, manual handling of objects (< 10 kg and ≥ 10 kg), time driving (for > 1 h), frequent bending, frequent twisting, frequent lifting, heavy lifting, running, and noncontact sports, were not significantly associated with the occurrence of flares within 24 h [11, 37].

### Pain intensity and disability

Although increased morning pain was associated with a higher risk of flares after 1 day (OR of 1.26 [1.05—1.50]) [12], disability was not significantly associated with the occurrence of flares over the subsequent 3 days.

### Sleep

Sleep quality showed a notable association with the risk of flares. In particular, “fairly bad” sleep quality was associated with a significantly increased risk of flares after 1 day (OR of 6.28 [2.09—18.81]), with even “fairly good” sleep quality being associated with increased risk of flares after 2 days (OR of 3.14 [1.25—7.93]) [12]. In contrast, objectively measured sleep duration was not significantly associated with flares over the subsequent 3 days [13].

### Psychological factors

Multiple psychological factors showed significant associations with flare risk: stress or depression (OR of 2.8 [1.2—6.7]) [11], mental distress (OR of 6.7 [2.5—17.5]) [37], and depressed mood (OR of 5.8 [2.6—12.8]) [37]. However, the association with depressed mood was inconsistent, with another study showing no significant association (OR of 1.2 [0.4—3.7]) [11]. Other psychological factors such as fear of physical activity, pain rumination, pain self-efficacy, and dissatisfaction with life were not significantly associated with flares [12, 37].

### Other factors

Physical therapy was associated with a reduced risk of flares within 24 h (OR of 0.4 [0.1—1.0]) [11]. Fatigue was not significantly associated with the occurrence of flares over the following 3 days [12].

### Patient perspectives on flare triggers

After surveying 130 individuals with LBP about their perceived flare triggers, a qualitative study by Costa et al. [29] found that the majority (84.8%) of the patients identified biomedical factors as the main triggers, notably active movements (35%), and static postures (28.1%). Specific triggers included bending, twisting, prolonged walking, strenuous exercise, daily tasks like gardening or vacuuming, lifting, prolonged sitting or standing, poor posture, and travel or driving. A small proportion (15.2%) of the patients reported that nonbiomedical factors, such as psychological stress, anxiety, weather conditions, sleep quality, diet, and fatigue, were the main triggers.

## Discussion

The current scoping review represents an important advancement in our understanding of flares in spinal and pelvic girdle pain. First, our findings revealed that the definition of flares has shifted beyond simply focusing on pain intensity to a more multidimensional concept that encompasses functional and emotional aspects. Second, although various tools have been used to assess flares, their reliability and validity have yet to be evaluated, emphasizing the need to develop a standardized measure with psychometric properties. Lastly, research on flare risk factors has been limited, with apparent gaps in the understanding of their diversity and complexity. Addressing these gaps is essential for developing effective prevention strategies and personalized treatments, making it a key challenge for future research.

### Definitions and domains of flares

Our review identified 15 studies that investigated the definitions and domains of flares in spinal and pelvic girdle pain [3, 4, 11–13, 27–34, 36, 37]. Notably, all of these studies focused exclusively on LBP, indicating a knowledge gap regarding flares in other conditions, such as neck pain and pelvic pain. However, recent evidence suggests that symptom trajectories among those with LBP may also apply to those with neck pain. For instance, Irgens et al. [10] revealed that neck pain shares similar symptom trajectories with LBP, including recurrent flare patterns. Although this similarity suggests common underlying mechanisms across different spinal regions, further research is needed to determine whether definitions for flares in LBP can be applied to those in other spine conditions or require condition-specific modifications.

The findings of the present scoping review reflect a shift in the conceptualization of LBP flares from a unidimensional, pain-centered model to a multidimensional framework that recognizes the complex interplay of biological, psychological, and social factors. This shift aligns with the broader trend in pain research toward comprehensive biopsychosocial models [38, 39]. Costa et al. [35] exemplified this progression through a multi-step process, defining a flare as “a worsening of your condition that lasts from hours to weeks, is difficult to tolerate, and generally impacts your usual activities and/or emotions,” highlighting three key domains, namely worsening of symptoms, impact on activities, and emotional impact.

However, despite this promising shift, no consensus has been reached regarding detailed definitions of individual domains constituting a flare and their relative importance in the experience of a flare. For instance, the symptom domain may include increased pain intensity, expanded areas of symptoms, various sensory changes (e.g., paresthesia), and muscle tension [30]. The activity domain encompasses limitations in daily tasks, reduced work productivity, and restricted social participation, highlighting the functional consequences of flares [30–32]. The emotional domain comprises various psychological responses, such as frustration, stress, anxiety, and mood disturbances [30, 31]. Although these findings can help characterize the multidimensional nature of flares, consensus through a Delphi study involving both experts on and individuals with spinal and pelvic girdle pain needs to be established given its importance for assessing flares and guiding the development of standardized assessment tools.

### Flare assessment tools

None of the included studies evaluated the reliability and validity of assessment tools for flares in spinal and pelvic girdle pain. Most of these studies used either generic pain measures, such as the NRS and VAS, or single-item self-report questionnaires to evaluate the presence or frequency of flares [3, 4, 11–13, 27, 28, 33, 34, 36, 37]. The use of unvalidated tools reflects a lack of consensus on the definition and domains of flares, which limits the precision and utility of current assessments.

The development of validated flare assessment tools could provide several important benefits. For instance, standardized assessment would enable systematic comparisons and meta-analyses, thereby strengthening available evidence for flare characteristics and interventions. Moreover, recent studies have shown that incorporating pain variability into traditional predictive models enhances outcome prediction accuracy [40]. Therefore, validated flare assessments may advance prognostic accuracy and support personalized treatment strategies. COSMIN guidelines [41, 42] can be useful for developing a validated assessment tool wherein content validity is verified first, followed by assessment of internal structures and finally the remaining measurement properties, such as reliability, measurement error, criterion validity, and hypotheses testing for construct validity and responsiveness. The current review can serve as the foundation for the verification of this first step.

### Risk factors and triggers

This review evaluated five studies that assessed risk factors and triggers for flares [11–13, 29, 37]. These studies consistently identified lifestyle factors as potential risk factors for flares. For example, three studies found that increased daily sitting time or sitting for over 1—6 h was associated with increased risk for flares [11, 12, 37]. Moreover, activities with a high total metabolic equivalent (MET) were identified as a protective factor that reduced the risk for flares [13]. This aligns with broader studies emphasizing the benefits of physical activity in managing musculoskeletal conditions [43–45]. Additionally, sleep quality and stress were also identified as factors affecting the risk for flares, with participants who reported “fairly poor” sleep quality having a significantly increased risk for flares [11, 12]. These findings reflect contemporary perspectives emphasizing lifestyle modifications in chronic pain management [46]. Notably, addressing modifiable factors could be a crucial strategy for the prevention and long-term management of flares.

Despite these findings, current evidence is quite limited in both scope and methodology. Several potential factors (e.g., nutritional factors, self-management abilities, social support, stress coping, and environmental factors) have remained unexplored. For example, evidence has shown that dietary patterns influence inflammation levels, which may potentially be a factor affecting the risk of flares [47]. Additionally, although the case-crossover design employed in most studies could be effective for controlling time-invariant confounders [11–13, 37], it may not capture the cumulative or long-term effects of these factors [48, 49]. Future research using longitudinal cohort designs to clarify the sustained impact of currently identified modifiable factors, such as physical activity, sleep quality, and stress, on the risk of flare occurrence, along with other lifestyle factors not yet examined, could further enhance our understanding of the mechanisms involved in the development of flares.

### Limitations

The present scoping review has several limitations. First, this review focused on studies explicitly using the term flare, and studies describing similar phenomena using related terms such as episode or recurrence may not have been captured. In particular, acute spinal pain studies may more commonly use such terms rather than flare, which may explain why most included studies focused on chronic LBP, and may have limited the exploration of how related terminology is used across different clinical contexts. However, this focus allowed us to clarify how the term flare has been used in the literature, providing a foundation for future research. Second, our search was limited to studies published in English, which may have overlooked relevant studies in other languages. However, evidence suggests that the inclusion of non-English studies in systematic reviews rarely changes the overall conclusions [50]. Additionally, methodological limitations of existing studies and the lack of validated assessment tools hindered our ability to draw firm conclusions regarding risk factors for flares. While a few risk factors such as prolonged sitting, prolonged standing, and psychological factors were evaluated in multiple studies, the majority of risk factors were assessed in only a single study, and all studies demonstrated a high RoB. Consequently, the overall certainty of evidence for risk factors was rated as very low in our GRADE assessment. Nevertheless, these limitations highlight areas in which future research can focus.

## Conclusions

The current scoping review aimed to clarify the current understanding of the definitions, assessment tools, and risk factors related to flares in spinal and pelvic girdle pain. Accordingly, our findings revealed that the definition of flares has shifted toward a multidimensional concept; however, this development has been limited to LBP, with no consensus having yet been reached on the specific domains that constitute flares. Current assessment methods lack validation and are limited to pain measures and single-item self-reports. Although potential risk factors include physical activity, sleep, and psychological factors, the evidence was limited by the high RoB and narrow scope of investigation. Future research should focus on establishing a consensus on flare domains, developing validated assessment tools, and conducting rigorous studies to identify a broader range of risk factors across spinal and pelvic girdle pain.

## Supplementary Information

Additional file 1. Preferred Reporting Items for Systematic reviews and Meta-Analyses extension for Scoping Reviews (PRISMA-ScR) checklist.

Additional file 2. Search strategy.

Additional file 3. Grading of Recommendations Assessment, Development and Evaluation (GRADE) assessment of the certainty of evidence for flare risk factor.

## Data Availability

All data extracted from included studies are available within the article and its supplementary materials. Additional extracted data are available from the corresponding author on reasonable request.

## Abbreviations

COSMIN: COnsensus-based Standards for the selection of health status Measurement Instruments
CI: Confidence interval
GRADE: Grading of Recommendations Assessment, Development and Evaluation
JBI: Joanna Briggs Institute
LBP: Low back pain
MET: Metabolic equivalent
NRS: Numerical rating scale
OR: Odds ratio
PRISMA-ScR: Preferred Reporting Items for Systematic Reviews and Meta-Analyses extension for Scoping Reviews
VAS: Visual analog scale
