# Supplementary material for: What do we know about flares in spinal and pelvic girdle pain? A scoping review
Source: BMC Musculoskelet Disord. 2026 Apr 28;27:516. doi: 10.1186/s12891-026-09884-w (PMC13274070; doi:10.1186/s12891-026-09884-w)
Supplement: Supplementary file 2 — Additional file 2. Search strategy. [file 12891_2026_9884_MOESM2_ESM.pdf]

Additional file 2. Search strategy.

MEDLINE

1. Neck Pain/
2. neck pain.ti,ab.
3. Brachial Plexus Neuritis/
4. cervico brachial neuralgia.ti,ab.
5. Headache/
6. headache.ti,ab.
7. cervicogenic headache.ti,ab.
8. neckache.ti,ab.
9. cervicalgia.ti,ab.
10. (spondylosis or spondylolysis).ti,ab.
11. Spinal osteophytosis/
12. Whiplash Injuries/
13. whiplash.ti,ab.
14. cervical pain.ti,ab.
15. cervicodynia.ti,ab.
16. brachial plexus neuritis.ti,ab.
17. rachialgia.ti,ab.
18. Radiculopathy/
19. Polyradiculopathy/
20. Neck Injuries/

21. neck injur*.ti,ab.
22. Torticollis/
23. cervicobrachial neuralgia.ti,ab.
24. Cervical Rib Syndrome/
25. dorsalgia.ti,ab.
26. exp Back Pain/
27. exp Back Injuries/
28. backache.ti,ab.
29. ((lumb* or back) adj pain).ti,ab.
30. coccyx.ti,ab.
31. coccydynia.ti,ab.
32. sciatica.ti,ab.
33. exp Sciatic Neuropathy/
34. spondylosis.ti,ab.
35. lumbago.ti,ab.
36. back disorder*.ti,ab.
37. (disc adj degeneration).ti,ab.
38. (disc adj prolapse).ti,ab.
39. (disc adj herniation).ti,ab.
40. (failed adj back).ti,ab.
41. exp Spinal Diseases/
42. exp Spine/
43. exp Spinal Cord Compression/
44. exp Spinal Cord Diseases/

45. (lumbar adj3 stenosis).ti,ab.
46. (spinal adj3 stenosis).ti,ab.
47. lateral root stenosis.ti,ab.
48. foraminal stenosis.ti,ab.
49. neurogenic claudication.ti,ab.
50. (facet adj arthrosis).ti,ab.
51. facet joint pain.ti,ab.
52. intervertebral disc disease.ti,ab.
53. myofascial pain.ti,ab.
54. nerve root inflammation.ti,ab.
55. myelopathy.ti,ab.
56. radiculalgia.ti,ab.
57. radiculitis.ti,ab.
58. (lumbar adj2 fusion).ti,ab.
59. Spinal Fusion/
60. Decompression, Surgical/
61. (nerve adj2 decompress*).ti,ab.
62. (root adj2 decompress*).ti,ab.
63. (fusion adj2 decompress*).ti,ab.
64. exp Spinal Dysraphism/
65. exp Spinal Fractures/
66. (thoracic adj3 pain).ti,ab.
67. (thoracic adj3 injur*).ti,ab.
68. thoracolumbar.ti,ab.

69. exp Pelvic Pain/
70. pelvic pain.ti,ab.
71. exp Lumbosacral Region/
72. exp Sacrococcygeal Region/
73. (sacral adj2 pain).ti,ab.
74. or/1-73
75. exp Symptom Flare Up/
76. flare*.ti,ab.
77. or/75-76
78. 74 and 77

Cochrane Central Register of Controlled Trials (CENTRAL)

1. MeSH descriptor: [Neck Pain] this term only
2. neck pain: ti,ab,kw
3. MeSH descriptor: [Brachial Plexus Neuritis] this term only
4. cervico brachial neuralgia: ti,ab,kw
5. MeSH descriptor: [Headache] this term only
6. Headache: ti,ab,kw
7. cervicogenic headache: ti,ab,kw
8. Neckache: ti,ab,kw
9. Cervicalgia: ti,ab,kw
10. (spondylosis OR spondylolysis): ti,ab,kw
11. MeSH descriptor: [Spinal osteophytosis] this term only
12. MeSH descriptor: [Whiplash Injuries] this term only

13. Whiplash: ti,ab,kw
14. cervical pain: ti,ab,kw
15. Cervicodynia: ti,ab,kw
16. brachial plexus neuritis: ti,ab,kw
17. Rachialgia: ti,ab,kw
18. MeSH descriptor: [Radiculopathy] this term only
19. MeSH descriptor: [Polyradiculopathy] this term only
20. MeSH descriptor: [Neck Injuries] this term only
21. neck injur*: ti,ab,kw
22. MeSH descriptor: [Torticollis] this term only
23. cervicobrachial neuralgia: ti,ab,kw
24. MeSH descriptor: [Cervical Rib Syndrome] this term only
25. Dorsalgia: ti,ab,kw
26. MeSH descriptor: [Back Pain] explode all trees
27. MeSH descriptor: [Back Injuries] explode all trees
28. Backache: ti,ab,kw
29. ((lumb* OR back) pain) : ti,ab,kw
30. Coccyx: ti,ab,kw
31. Coccydynia: ti,ab,kw
32. Sciatica: ti,ab,kw
33. MeSH descriptor: [Sciatic Neuropathy] explode all trees
34. Spondylosis: ti,ab,kw
35. Lumbago: ti,ab,kw
36. back disorder*: ti,ab,kw

37. disc degeneration: ti,ab,kw

38. disc prolapse: ti,ab,kw

39. disc herniation: ti,ab,kw

40. failed back: ti,ab,kw

41. MeSH descriptor: [Spinal Diseases] explode all trees

42. MeSH descriptor: [Spine] explode all trees

43. MeSH descriptor: [Spinal Cord Compression] explode all trees

44. MeSH descriptor: [Spinal Cord Diseases] explode all trees

45. (lumbar NEAR/3 stenosis): ti,ab,kw

46. (spinal NEAR/3 stenosis): ti,ab,kw

47. lateral root stenosis: ti,ab,kw

48. foraminal stenosis: ti,ab,kw

49. neurogenic claudication: ti,ab,kw

50. facet arthrosis: ti,ab,kw

51. facet joint pain: ti,ab,kw

52. intervertebral disc disease: ti,ab,kw

53. myofascial pain: ti,ab,kw

54. nerve root inflammation: ti,ab,kw

55. myelopathy: ti,ab,kw

56. radiculalgia: ti,ab,kw

57. radiculitis: ti,ab,kw

58. (lumbar NEAR/2 fusion) : ti,ab,kw

59. MeSH descriptor: [Spinal Fusion] this term only

60. MeSH descriptor: [Decompression, Surgica] this term only

61. (nerve NEAR/2 decompress*): ti,ab,kw
62. (root NEAR/2 decompress*): ti,ab,kw
63. (fusion NEAR/2 decompress*): ti,ab,kw
64. MeSH descriptor: [Spinal Dysraphism] explode all trees
65. MeSH descriptor: [Spinal Fractures] explode all trees
66. (thoracic NEAR/3 pain): ti,ab,kw
67. (thoracic NEAR/3 injur*): ti,ab,kw
68. Thoracolumbar: ti,ab,kw
69. MeSH descriptor: [Pelvic Pain] explode all trees
70. pelvic pain: ti,ab,kw
71. MeSH descriptor: [Lumbosacral Region] explode all trees
72. MeSH descriptor: [Sacrococcygeal Region] explode all trees
73. (sacral NEAR/2 pain): ti,ab,kw
74. or/1-73
75. MeSH descriptor: [Symptom Flare Up] explode all trees
76. flare*: ti,ab,kw
77. or/75-76
78. 74 and 77

EMBASE

1. exp Neck Pain/
2. neck pain.ti,ab.
3. exp Brachial Plexus Neuritis/
4. cervico brachial neuralgia.ti,ab.

5. exp Headache/
6. headache.ti,ab.
7. cervicogenic headache.ti,ab.
8. neckache.ti,ab.
9. cervicalgia.ti,ab.
10. spondylosis OR spondylolysis.ti,ab.
11. exp Spinal osteophytosis/
12. exp Whiplash Injuries/
13. whiplash.ti,ab.
14. cervical pain.ti,ab.
15. cervicodynia.ti,ab.
16. brachial plexus neuritis.ti,ab.
17. rachialgia.ti,ab.
18. exp Radiculopathy/
19. exp Polyradiculopathy/
20. exp Neck Injuries/
21. neck injur*.ti,ab.
22. exp Torticollis/
23. cervicobrachial neuralgia.ti,ab.
24. exp Cervical Rib Syndrome/
25. dorsalgia.ti,ab.
26. exp Back Pain/
27. exp Back Injuries/
28. backache.ti,ab.

29. (lumb* OR back) pain.ti,ab.
30. coccyx.ti,ab.
31. coccydynia.ti,ab.
32. sciatica.ti,ab.
33. exp Sciatic Neuropathy/
34. spondylosis.ti,ab.
35. lumbago.ti,ab.
36. back disorder*.ti,ab.
37. disc degeneration.ti,ab.
38. disc prolapse.ti,ab.
39. disc herniation.ti,ab.
40. failed back.ti,ab.
41. exp Spinal Diseases/
42. exp Spine/
43. exp Spinal Cord Compression/
44. exp Spinal Cord Diseases/
45. lumbar stenosis.ti,ab.
46. spinal stenosis.ti,ab.
47. lateral root stenosis.ti,ab.
48. foraminal stenosis.ti,ab.
49. neurogenic claudication.ti,ab.
50. facet arthrosis.ti,ab.
51. facet joint pain.ti,ab.
52. intervertebral disc disease.ti,ab

53. myofascial pain.ti,ab.
54. nerve root inflammation.ti,ab.
55. myelopathy.ti,ab.
56. radiculalgia.ti,ab.
57. radiculitis.ti,ab.
58. lumbar fusion.ti,ab.
59. exp Spinal Fusion/
60. exp Decoti,abression, Surgical/
61. nerve decompress*.ti,ab.
62. root decompress*.ti,ab.
63. fusion decompress*.ti,ab.
64. exp Spinal Dysraphism/
65. exp Spinal Fractures/
66. thoracic pain.ti,ab.
67. thoracic injur*.ti,ab.
68. thoracolumbar.ti,ab.
69. exp Pelvic Pain/
70. pelvic pain.ti,ab.
71. exp Lumbosacral Region/
72. exp Sacrococcygeal Region/
73. sacral pain.ti,ab.
74. or/1-73
75. flare* ti,ab.
76. 74 and 75

CINAHL

1. (MH Neck Pain)
2. neck pain
3. (MH Brachial Plexus Neuritis)
4. cervico brachial neuralgia
5. (MH Headache)
6. headache
7. cervicogenic headache
8. neckache
9. cervicalgia
10. (spondylosis OR spondylolysis)
11. (MH Spinal osteophytosis)
12. (MH Whiplash Injuries)
13. whiplash
14. cervical pain
15. cervicodynia
16. brachial plexus neuritis
17. rachialgia
18. (MH Radiculopathy)
19. (MH Polyradiculopathy)
20. (MH Neck Injuries)
21. neck injur*
22. (MH Torticollis)

23. cervicobrachial neuralgia
24. (MH Cervical Rib Syndrome)
25. dorsalgia
26. (MH Back Pain+)
27. (MH Back Injuries+)
28. backache
29. ((lumb* OR back) N1 pain)
30. coccyx
31. coccydynia
32. sciatica
33. (MH Sciatic Neuropathy+)
34. spondylosis
35. lumbago
36. back disorder*
37. (disc N1 degeneration)
38. (disc N1 prolapse)
39. (disc N1 herniation)
40. (failed N1 back)
41. (MH Spinal Diseases+)
42. (MH Spine+)
43. (MH Spinal Cord Compression+)
44. (MH Spinal Cord Diseases+)
45. (lumbar N3 stenosis)
46. (spinal N3 stenosis)

47. lateral root stenosis
48. foraminal stenosis
49. neurogenic claudication
50. (facet adj arthrosis)
51. facet joint pain
52. intervertebral disc disease
53. myofascial pain
54. nerve root inflammation
55. myelopathy
56. radiculalgia
57. radiculitis
58. (lumbar N2 fusion)
59. (MH Spinal Fusion)
60. (MH Decompression, Surgical)
61. (nerve N2 decompress*)
62. (root N2 decompress*)
63. (fusion N2 decompress*)
64. (MH Spinal Dysraphism+)
65. (MH Spinal Fractures+)
66. (thoracic N3 pain)
67. (thoracic N3 injur*)
68. Thoracolumbar
69. (MH Pelvic Pain+)
70. pelvic pain

71. (MH Lumbosacral Region+)

72. (MH Sacrococcygeal Region+)

73. (sacral N2 pain)

74. or/1-73

75. (MH Symptom Flare Up+)

76. flare*

77. or/75-76

78. 74 and 77

Web of science

TS=(((neck pain) OR (headache) OR (nechache) OR (cervicalgia) OR (spondylosis) OR (spinal osteophytosis) OR (whiplash) OR (cervical pain) OR (cervicornia) OR (rachialgias) OR (radiculopathy) OR (polyradiculopathy) OR (neck injur*) OR (torticollis) OR (cervicobrachial neuralgia) OR (cervical rib syndrome) OR (dorsalia) OR (back pain) OR (back injur*) OR (backache) OR (coccyx) OR (coccydynia) OR (sciatica) OR (sciatic neuropathy) OR (spondylosis) OR (lumbago) OR (back disorder*) OR (disc herniation) OR (spinal diseases) OR (lumbar stenosis) OR (intervertebral disc disease) OR (myofascial pain) OR (myelopathy) OR (radiculalgic) OR (lumbar fusion) OR (spinal fractures) OR (thoracic pain) OR (thoracic injur*) OR (pelvic pain) OR (sacral pain)) AND (flare*))
