# Supplementary material for: What do we know about flares in spinal and pelvic girdle pain? A scoping review
Source: BMC Musculoskelet Disord. 2026 Apr 28;27:516. doi: 10.1186/s12891-026-09884-w (PMC13274070; doi:10.1186/s12891-026-09884-w)
Supplement: Supplementary file 3 — Additional file 3. Grading of Recommendations Assessment, Development and Evaluation (GRADE) assessment of the certainty of evidence for flare risk factor. [file 12891_2026_9884_MOESM3_ESM.pdf]

Additional file 3. Grading of Recommendations Assessment, Development and Evaluation (GRADE) assessment of the certainty of the evidence for risk factors for flares.

Risk factors	Number of particip ants	Number of studies	Number of cohorts	Phase	Study limitatio ns	Inconsis tency	Indirect ness	Impreci sion	Publicat ion bias	Overall quality
Prolonged sitting	286	3	3	1	X	✓	✓	✓	X	+
Prolonged standing	286	3	3	1	X	X	✓	✓	X	+
Prolonged walking	238	2	2	1	X	X	✓	✓	X	+
Lifting a heavy object	200	2	2	1	X	✓	✓	✓	X	+
Frequent lifting	152	1	1	1	X	X	✓	✓	X	+
Frequent bending	152	1	1	1	X	X	✓	✓	X	+
Frequent twisting	152	1	1	1	X	X	✓	✓	X	+

Running or jogging	48	1	1	1	X	X	✓	X	X	+
Non-contact sports	48	1	1	1	X	X	✓	X	X	+
Time driving	152	1	1	1	X	X	✓	✓	X	+
Transport	86	1	1	1	X	X	✓	X	X	+
Leisure	86	1	1	1	X	X	✓	X	X	+
Work	86	1	1	1	X	X	✓	X	X	+
%MET 3-4	86	1	1	1	X	X	✓	X	X	+
%MET >4	86	1	1	1	X	X	✓	X	X	+
Pain	86	1	1	1	X	X	✓	X	X	+
Disability	86	1	1	1	X	X	✓	X	X	+
Sleep quality	86	1	1	1	X	X	✓	X	X	+
Sleep rate	86	1	1	1	X	X	✓	X	X	+
Sleep hours	86	1	1	1	X	X	✓	X	X	+
In-bed hours	86	1	1	1	X	X	✓	X	X	+
Bed time	86	1	1	1	X	X	✓	X	X	+

Wake time	86	1	1	1	X	X	✓	X	X	+
Depressed mood	200	2	2	1	X	X	✓	✓	X	+
Mental distress	152	1	1	1	X	X	✓	✓	X	+
Stress or depression	48	1	1	1	X	X	✓	X	X	+
Stressful event	48	1	1	1	X	X	✓	X	X	+
Dissatisfaction with life	152	1	1	1	X	X	✓	✓	X	+
Rumination	86	1	1	1	X	X	✓	X	X	+
Fear of physical activity	86	1	1	1	X	X	✓	X	X	+
Pain self-efficacy	86	1	1	1	X	X	✓	X	X	+
Physical therapy	48	1	1	1	X	X	✓	X	X	+
Treatment	86	1	1	1	X	X	✓	X	X	+
Medication	86	1	1	1	X	X	✓	X	X	+

Fatigue	86	1	1	1	X	X	✓	X	X	+

Abbreviations: MET, Metabolic equivalent.

GRADE factors: ✓, no serious limitations; X, serious limitations.

For overall quality of evidence: +, very low; ++, low; +++, moderate; ++++, high.
